# Effects of anti-inflammatory vagus nerve stimulation on the cerebral microcirculation in endotoxinemic rats

**DOI:** 10.1186/1742-2094-9-183

**Published:** 2012-07-25

**Authors:** Stanka Mihaylova, Anke Killian, Konstantin Mayer, Soni Savai Pullamsetti, Ralph Schermuly, Bernhard Rosengarten

**Affiliations:** 1 Department of Neurology, Justus Liebig University, Klinikstrasse 33, D-35392, Giessen, Germany; 2 Department of Internal Medicine, Justus Liebig University, Klinikstrasse 36, D-35392, Giessen, Germany; 3 Department of Lung Development and Remodeling, Max Planck Institute for Heart and Lung Research, Ludwigstrasse 43, D-61231, Bad Nauheim, Germany

**Keywords:** Microcirculation, Brain, Neurovascular coupling, Evoked potentials, Hypoxia, Sepsis, Inflammation, Lipopolysaccharide, EEG, Vagus nerve stimulation

## Abstract

**Background:**

In sepsis syndromes the severity of the inflammation triggers microvascular dysfunction and early organ failure. We studied the effects of anti-inflammatory vagus nerve stimulation on the cerebral microcirculatory integrity in an endotoxinemic rat model.

**Methods:**

In both control and endotoxinemic (5 mg/kg lipopolysaccharide i.v.) rats, the effect of cervical bilateral vagotomy with or without left-sided distal vagus nerve stimulation were compared to non-vagotomized, nonstimulated group (sham). Neurovascular coupling was analyzed by electrical forepaw stimulation, EEG, and cortical laser-Doppler flow recording. Resting cerebral blood flow, evoked potentials and hemodynamic responses, were obtained over a period of 4.5 hours. Regulation of the nitric oxide system (iNOS expression and nitrite/nitrate measurements), cytokines (IFN-γ, TNF-α, IL-6, IL-10), hypoxic and apoptosis signaling molecules (HIF-2α, Bax) were measured at the end of experiments.

**Results:**

In endotoxinemic rats, vagus nerve stimulation tended to increase anti-inflammatory cytokine levels and resulted in a stabile hemodynamic response (28 ± 13%; versus baseline). Vagotomized animals incurred a pro-inflammatory response (7 ± 4%; *P* < 0.0001 versus baseline) and produced more HIF-2α than vagotomized vagus nerve stimulated (VNS) animals. Evoked potential amplitudes were stabilized in VNS (15 ± 7 μV; n.s. versus baseline) as compared to vagotomised rats (8 ± 5 μV; *P* < 0.001 versus baseline). However, no effects were observed on apoptosis markers or nitric oxide levels.

**Conclusions:**

Vagus nerve stimulation in endotoxinemic rats had a positive effect on neurovascular coupling and stabilized evoked potentials.

## Background

Sepsis is the most frequent cause of death in intensive care units (ICUs). In industrialized countries, it has an average incidence of 300/100,000 per year, increasing significantly in the elderly [1,2]. Despite many improvements in the therapy of ICU patients, mortality from sepsis remains high. Each year, in the United States alone, approximately 600,000 patients develop sepsis; resulting in the death of approximately 240,000 people [3].

Sepsis is increasingly recognized as an acute disease process as treatment during the first six to twenty-four hours is critical for patient survival [4]. The brain is one of the first organ systems affected by sepsis. A sepsis-associated delirium occurs in 30% to 70% of cases, often preceding other signs of sepsis [5,6]. Neurovascoular coupling (NVC) adapts blood–brain circulation in accordance with blood–brain demands. NVC, therefore, adapts to disturbances in metabolically driven blood flow, which leads to changes in neurofunctional parameters [7]. In addition to cytokine storms, nitric oxide (NO) production by inducible nitric oxide synthase (iNOS) is generally assumed to play an important role in early microcirculatory failure, which triggers further organ dysfunction [7-13].

Distal vagus nerve stimulation (VNS) was shown to mitigate the inflammatory response and thereby improve survival in a variety of animal sepsis models [14,15]. An inhibition of the inflammatory response reduces cytokine levels and lowers the induction of the iNOS [16]. Inhibition of iNOS by the specific inhibitor 1400W improved microvascular function in the brain in endotoxinemic rats [8]. However, possible effects on the nitric oxide system, and therefore microcirculatory functioning, have not been investigated under VNS.

In this study, we investigated the role of distal VNS versus vagotomy (VGX) on the integrity of the NVC in the brain of endotoxinemic rats by laser Doppler flowmetry (LDF). We also monitored the effects that VNS had on cytokines, apoptosis, hypoxia, and the NO system. To assess possible nonspecific effects of either VNS or VGX on NVC, corresponding experiments were performed on nonseptic rats.

## Methods

All animal experiments were performed in strict accordance with the National Institutes of Health Guide for Care and Use of Laboratory Animals and approved by the local Animal Care and Use Committee of the ‘Regierungspräsidium’ Giessen, Hessen, Germany.

### General preparation

Adult male Sprague Dawley rats (290 to 320 g) were purchased from Harlan Laboratories (Harlan, Rossdorf, Germany). They were housed five to a cage with food and water available *ad libitum* and were maintained on a 12-hour light/dark cycle (lights on at 07:00). For the experiments, rats were initially anesthetized with isoflurane, tracheotomized, paralyzed with pancuronium bromide (0.2 mg/kg/h) and mechanically ventilated (Harvard Rodent Ventilator; Harvard Apparatus, South Natick, MA, USA). The right femoral artery and vein were cannulated for blood pressure recording, blood sampling, and drug administration. Rectal body temperature was maintained at 37°C using a feedback-controlled heating pad.

Isoflurane anesthesia was discontinued and replaced by an intravenous application of α-chloralose (80 mg/kg; Sigma-Aldrich Chemie GmbH, Taufkirchen, Germany) for a period of 60 minutes to washout isoflurane. α-chloralose anesthesia was continued for the duration of the experiment. Supplementary doses of chloralose (30 mg/kg) were given every hour. During chloralose anesthesia, the animals were ventilated with a 1:1 mixture of nitrogen and oxygen. Arterial blood gas analyses and pH were measured repeatedly at least every 30 minutes (blood gas analyzer model Rapidlab 348; Bayer Vital GmbH, Fernwald, Germany). Glucose and lactate levels were measured simultaneously (Glukometer Elite XL, Bayer Vital GmbH, Fernwald, Germany; Lactate pro, Arkray Inc. European Office, Düsseldorf, Germany), while glucose concentrations were maintained at > 60 mg/dl. A moderate volume therapy of 1.2 ml/hour of 0.9% NaCl was given to replace renal and perspirative fluid losses.

### Neurovascular coupling measurements

The head of each animal was fixed in a stereotaxic frame without perforating the tympanum. The apex of the skull was exposed, and the bone over the left parietal cortex was thinned with a saline-cooled drill to allow transcranial laser Doppler flowmetry (LDF). The laser probe (BRL-100; Harvard Apparatus, South Natick, MA, USA) was placed 3.5 mm lateral and 1 mm rostral to the bregma in accordance with the coordinates of the somatosensory cortex, a location that corresponds closely to the region of maximal hemodynamic response during contralateral forepaw stimulation. The laser Doppler signal and the systemic mean arterial blood pressure were recorded continuously and processed on a personal computer running data acquisition software (Neurodyn; HSE, March-Hugstetten, Germany). Since the laser Doppler measures flow changes rather than absolute values, resting LDF signals had arbitrary units. However, evoked signal changes were used to assess flow changes, which are given in percentage changes from the baseline.

Brain electrical activity was recorded monopolarily with an active calomel electrode placed 0.5 mm behind the laser probe and an indifferent calomel electrode placed on the nasal bone. Signals were recorded and amplified (BPA Module 675; HSE, March-Hugstetten, Germany) and somatosensory evoked potentials (SEP) were averaged using the Neurodyn acquisition software (HSE, March-Hugstetten, Germany).

Somatosensory stimulation was achieved with electrical pulses applied by small needle electrodes inserted under the skin of the right forepaw (PSM Module 676; HSE, March-Hugstetten, Germany). The pulses consisted of rectangular bipolar pulses of 1.5 mA and a pulse width of 0.3 ms. The pulse repetition frequency was set at 2 Hz and stimulations were carried out for 30 seconds. Stimulation with 1.5 mA ensured that pain fibers were unaffected, thereby preventing changes in blood pressure. A rest of 30 seconds followed each stimulation train while activation rest cycles were repeated 10 times in order to increase the signal to noise ratio.

Flow velocity responses were averaged and calculated relative to the resting phase, which was set to zero. The evoked flow velocity responses (EFVR) were calculated from the averaged relative flow velocity signals under conditions of stimulation.

Evoked potential amplitudes were calculated from the N2-P1 amplitude differences and the latency between the beginning of stimulation and the occurrence of the P1 peak was examined.

### Vagus nerve stimulation

**Figure 1:**
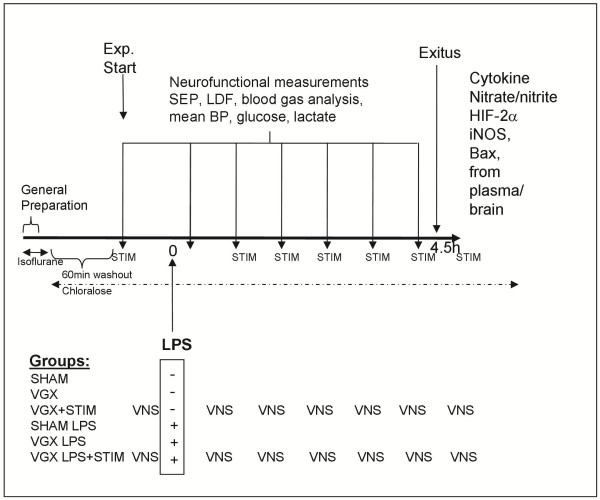
Illustration of the preparation period and experimental protocol together with time points of vagal interaction or neurofunctional measurements.

The nervus vagus of each side was exposed at the cervical level lateral of the trachea running in parallel with the common carotid artery. Both vagus nerves were carefully dissected from their artery in each rat. The right vagus nerve mainly innervates the thoracic organs such as the lung and heart, whereas the left trunk mainly innervates the intestinal organs and the plexus mesentericus. For vagus nerve stimulation, the left nerve was cut. The distal part of the nerve was then fixed in a special nerve stimulation clamp (HSE, March-Hugstetten, Germany) to allow electrical stimulation of the distal trunk of the vagus nerve. To exclude aberrant innervations of the intestinal organs from the right nervus vagus, this nerve was also cut. Electrical pulses of 2 mA were applied during a 0.3 ms pulse width, and 2 Hz repetition frequency for a period of 10 minutes [15]. Stimulation blocks were repeated every 45 minutes, starting before the application of endotoxin and continuing until the end of experimentation (4.5 hours). Additional experiments were performed to measure neurovascular coupling (Figure 1).

### Study design

In random order, 60 rats received 5 mg/kg body weight intravenous lipopolysaccharide (LPS, from *Escherichia coli*, O111:B4; Sigma-Aldrich Chemie GmbH, Germany) or 0.5 ml of 0.9% NaCl. LPS was dissolved in 0.5 ml of 0.9% NaCl and slowly administered for 5 minutes. Endotoxinemic and sham groups were further divided into three subgroups. In one subgroup, bilateral cervical vagotomy (VGX) was performed without stimulation of the nerve (LPS group: VGX LPS; nonseptic group: VGX). In a second group, the distal trunk of the left vagus nerve was stimulated after bilateral cervical vagotomy (LPS group: VGX LPS + STIM; sham group: VGX + STIM). In a third group, the operation of the vagus nerve was stopped before vagotomy and the vagus nerve was not stimulated (LPS group: SHAM LPS; nonseptic group: SHAM). Each experimental group consisted of 10 animals. Experiments were performed up to 4.5 hours after LPS/vehicle administration since previous studies have shown that blood pressure levels remain above the lower limit of the cerebral autoregulation during this time period [7,8], thus excluding secondary ischemic brain damage due to a decline in systemic blood pressure. Plasma samples were obtained before the rats were killed. Brains were removed and stored at −80°C.

### Biochemical determination of the effects of VNS or VGX on inflammatory parameters

The following experiments were carried out on the endotoxinemic groups (SHAM LPS, VGX LPS, VGX LPS + STIM) and the non-treated control group (SHAM).

### Immunoblotting

Rat cortex tissues obtained from three animals from each group were lysed in RIPA buffer containing Na_3_VO_4_, protease inhibitors, and PMSF. The lysates were resolved with SDS-PAGE (10% acrylamide) and transferred onto nitrocellulose membranes, blocked for one hour in 5% non-fat milk at room temperature and probed with the appropriate primary antibody overnight at 4°C at the following dilutions: β-actin (Abcam plc, Cambridge, United Kingdom; ab6276,1:3000), iNOS (Santa Cruz Biotechnology, Heidelberg, Germany; iNOS (M-19) sc-650,1:800), HIF-2α (Novus Biologicals, Cambridge, United Kingdom; NB100-132,1:500). After overnight incubation in primary antibody, membranes were washed with wash buffer and incubated with the respective horseradish peroxidase-conjugated polyclonal secondary antibodies for one hour at room temperature (RT). After washing, the blots were developed with an enhanced chemiluminescence (ECL) kit (GE Healthcare Europe GmbH, Freiburg, Germany) followed by film exposure. The reported optical densities are the means and SEMs of three separate experiments.

### RNA isolation, real-time PCR (RT-PCR), and quantitative real-time PCR (qRT-PCR)

Total RNA was extracted from frozen rat cortex tissue using Trizol reagent (Invitrogen, Carlsbad, CA, USA) following the manufacturer’s protocols. Each group consisted of n = 6 rats. cDNA synthesis was performed with the ImProm-II™ Reverse Transcription System (Promega, Madison, WI, USA) by incubating 0.8 μg of RNA according to the manufacturer’s instructions. qRT-PCR was performed with 2 μl cDNA. The oligonucleotide primer pairs (rat origin) were as follows: rat porphobilinogen deaminase (PBGD) 5′-CAAGGTTTTCAGCATCGCTACCA-3′ (forward) and 5′-ATGTCCGGTAACGGCGGC-3′ (reverse); rat inducible nitric oxide synthase (iNOS) 5′-CAGCCACCTTGGTGAGGGGA-3′ (forward) and 5′- TCCAGGGGCAAGCCATGTCT-3′ (reverse); rat Bcl-2-associated X protein (BAX) 5′-CAAGAAGCTGAGCGAGTGTCT -3′ (forward) and 5′-CAATCATCCTCTGCAGCTCCATATT-3′ (reverse).

qRT-PCR was carried out in a Stratagene Mx3000P™ qPCR system (Stratagene, La Jolla, CA, USA) and data were analyzed with accompanying software. The cycling protocol was as follows: polymerase activation, 95°C for 10 minutes; 40 cycles with denaturation at 95°C for 30 seconds, annealing at 58°C for 30 seconds and extension at 72°C for 30 seconds. To ensure that a single, specific product was generated, dissociation curves were evaluated. Relative expression levels were calculated as ΔCt values by normalizing Ct values of target genes to Ct values of PBGD as previously described. For every gene we performed three independent measurements. All data reported are the means and SEM of three separate experiments.

### Cytokine ELISA

Following the experiments, blood samples were drawn into tubes containing heparin sodium 2500 (Ratiopharm, Ulm, Germany). They were immediately centrifuged and separated, and the plasma stored at −80°C until analysis. Cytokine determinations for IFN-γ, TNF-α, IL-6, IL-10 concentrations in plasma samples were performed with ELISA sets (BD Bioscience, Heidelberg, Germany) for each LPS group (n = 6) as well as for the SHAM group.

### Measurement of NO concentration in plasma samples

NO metabolite (nitrite and nitrate) concentrations were determined for each of 10 samples in the sepsis groups and in the SHAM group using NOA Sievers 280 (FMI GmbH, Seeheim, Germany) according to the manufacturer’s instructions. Briefly, plasma samples were drawn 4.5 hours after LPS injection into heparin-containing tubes, immediately centrifuged, and stored at −20°C until measurements were taken. NO reaction products in samples were reduced by vanadium chloride. Resulting gaseous NO was detected by NOA Sievers 280, which was connected to a computer for data transfer and analysis by NOAWIN 32 software (DeMeTec, Langgöns, Germany).

### Statistical evaluation of physiological data

Statistical analyses were performed with a two-way ANOVA with the factors within and between groups to assess differences between the groups. In cases of significance, a Scheffe’s post hoc test was applied. For intergroup comparisons the statistical analyses were performed against baseline measurements. If the assumptions of normal distribution and equality of variances could not be assured in the statistic F-test, a nonparametric paired-sign or Kolmogorov-Smirnov test was conducted (Statview, SAS, Cary, NC, USA). The significance level was set to *P* < 0.05.

## Results

### General findings

The SHAM group exhibited stable blood gas, hemodynamic and neurofunctional parameters throughout the study (Tables 1 and 2). The higher pH at baseline was related to the α-chloralose which leads to a well-known metabolic alkalosis. Due to the administration volume there was a significant decrease in the hemoglobin concentration at the end of the study as compared to the baseline (140 ± 10 g/l versus120 ± 20 g/l; *P* < 0.001).

**Table 1:** Group averaged data for glucose, lactate, pH, pC02 and hemoglobin

	**Glucose (mg/dl)**		**Lactate (mmol/l)**		**pH**		**p0**_**2**_**(mmHg)**		**pC0**_**2**_**(mmHg)**		**Hemoglobin (g/l)**	
	**Baseline**	**End**	**Baseline**	**End**	**Baseline**	**End**	**Baseline**	**End**	**Baseline**	**End**	**Baseline**	**End**
SHAM	63 ± 8	84 ± 21	0.8 ± 0.05	0.9 ± 0.1	7.49 ± 0.06	7.47 ± 0.03	250 ± 18	246 ± 29	36 ± 3	38 ± 4	140 ± 10	120 ± 20**
VGX	98 ± 30	98 ± 18	0.8 ± 0.03	0.9 ± 0.1	7.45 ± 0.05	7.50 ± 0.06	265 ± 19	250 ± 23	41 ± 8	33 ± 4	140 ± 10	130 ± 10*
VGX + STIM	71 ± 15	105 ± 49	0.9 ± 0.2	1.3 ± 0.5	7.52 ± 0.06	7.51 ± 0.04	259 ± 19	248 ± 5	37 ± 5	36 ± 5	130 ± 10	100 ± 20*
SHAM LPS	65 ± 13	69 ± 26	0.8 ± 0.1	1.9 ± 0.5^s^	7.47 ± 0.05	7.45 ± 0.03	250 ± 14	233 ± 30	40 ± 4	35 ± 3*	150 ± 10	120 ± 10 ^s^
VGX LPS	82 ± 22	129 ± 90	0.8 ± 0.1	2.2 ± 0.7^s^	7.45 ± 0.07	7.37 ± 0.08**	272 ± 13	249 ± 26	41 ± 6	39 ± 4	150 ± 10	120 ± 20**
VGX LPS+ STIM	85 ± 25	65 ± 20	0.9 ± 0.1	1.8 ± 0.6***	7.48 ± 0.4	7.43 ± 0.1	251 ± 22	233 ± 21	38 ± 2	35 ± 5	130 ± 10	100 ± 20***

Data are given as the mean ± standard deviation (SD) together with statistical differences. Significance is given as: *, *P* < 0.05; **, *P* < 0.01; ***, *P* < 0.001; ^s^, *P* < 0.0001 compared with baseline. LPS, lipopolysaccharide; pC0_2_, partial pressure of carbon dioxide; SHAM, sham surgery; VGX, bilateral vagotomy; VGX + STIM, bilateral vagotomy and distal vagus nerve stimulation.

**Table 2:** Group averaged data for mean blood pressure, somatosensory evoked potentials, P1 latencies, evoked flow velocity responses and resting LDF signal at different time points of the experiment

		**Baseline**	**30 min**	**60 min**	**120 min**	**180 min**	**240 min**	**270 min**
Mean BP (mmHg)	SHAM	116 ± 20	120 ± 12	108 ± 20	113 ± 14	114 ± 19	108 ± 20	108 ± 20
Mean BP (mmHg)	VGX	115 ± 13	110 ± 13	112 ± 19	109 ± 14	110 ± 17	115 ± 22	115 ± 22
Mean BP (mmHg)	VGX + STIM	109 ± 10	99 ± 14	103 ± 18	100 ± 23	104 ± 29	93 ± 21	93 ± 21
Mean BP (mmHg)	SHAM LPS	123 ± 11	118 ± 14	106 ± 15**	104 ± 10**	90 ± 12^s^	90 ± 18^s^	90 ± 18^s^
Mean BP (mmHg)	VGX LPS	117 ± 9	109 ± 17	91 ± 17^s^	94 ± 18***	101 ± 13*	97 ± 10**	97 ± 10**
Mean BP (mmHg)	VGX LPS + STIM	119 ± 13	114 ± 12	94 ± 17*	92 ± 7***	86 ± 20 ^s^	90 ± 14***	90 ± 14***
SEP (μV)	SHAM	13 ± 7	15 ± 6	14 ± 4	15 ± 5	14 ± 5	15 ± 5	14 ± 5
SEP (μV)	VGX	15 ± 9	15 ± 8	16 ± 9	16 ± 9	15 ± 6	15 ± 6	16 ± 8
SEP (μV)	VGX + STIM	15 ± 9	13 ± 4	14 ± 4	12 ± 5	11 ± 5	13 ± 3	15 ± 8
SEP (μV)	SHAM LPS	17 ± 5	16 ± 6	15 ± 6	12 ± 6	11 ± 5	11 ± 4**	10 ± 6**
SEP (μV)	VGX LPS	18 ± 6	15 ± 4	14 ± 5	12 ± 4**	11 ± 7**	10 ± 5**	8 ± 5***
SEP (μV)	VGX LPS + STIM	18 ± 8	18 ± 9	19 ± 9	16 ± 5	16 ± 5	14 ± 4	15 ± 7
P1 latency (ms)	SHAM	10.8 ± 1	11.1 ± 1	11.2 ± 1	11.1 ± 1	11.3 ± 1	11.4 ± 1	11.7 ± 2
P1 latency (ms)	VGX	10 ± 0.6	10 ± 0.5	10 ± 0.6	10 ± 0.4	10 ± 0.4	10 ± 0.4	10.1 ± 0.5
P1 latency (ms)	VGX + STIM	10.2 ± 1	10.4 ± 1	10.5 ± 1	10.3 ± 1	10 ± 0.7	10 ± 0.7	10 ± 0.9
P1 latency (ms)	SHAM LPS	10 ± 0.5	10 ± 0.6	10 ± 0.5	10 ± 0.6	10 ± 0.6	10.3 ± 0.7	10.4 ± 1
P1 latency (ms)	VGX LPS	10 ± 0.8	10.4 ± 1	10.4 ± 0.8	10.6 ± 0.4	10.6 ± 0.7	11 ± 0.9	11 ± 1
P1 latency (ms)	VGX LPS + STIM	10 ± 0.4	10 ± 0.3	10 ± 0.3	10 ± 0.3	10 ± 0.4	10.2 ± 0.4	10 ± 0.4
EFVR (%)	SHAM	34 ± 10	26 ± 7	30 ± 11	31 ± 15	29 ± 14	31 ± 12	28 ± 7
EFVR (%)	VGX	23 ± 13	23 ± 15	21 ± 11	24 ± 15	24 ± 6	23 ± 7	24 ± 11
EFVR (%)	VGX + STIM	33 ± 13	35 ± 18	36 ± 13	29 ± 17	31 ± 18	32 ± 25	38 ± 14
EFVR (%)	SHAM LPS	27 ± 11	29 ± 11	26 ± 8	18 ± 10	18 ± 16	14 ± 11*	12 ± 8*
EFVR (%)	VGX LPS	34 ± 9	36 ± 7	26 ± 5	19 ± 9**	17 ± 16**	7 ± 5^s^	7 ± 4^s^
EFVR (%)	VGX LPS + STIM	34 ± 8	35 ± 11	32 ± 9	31 ± 13	28 ± 12	29 ± 13	28 ± 13
Resting LDF (U)	SHAM	163 ± 66	152 ± 71	140 ± 62	145 ± 82	142 ± 75	145 ± 71	139 ± 72
Resting LDF (U)	VGX	233 ± 78	219 ± 106	211 ± 88	199 ± 84	203 ± 101	213 ± 111	201 ± 107
Resting LDF (U)	VGX + STIM	224 ± 92	231 ± 75	232 ± 60	239 ± 30	193 ± 33	196 ± 49	186 ± 57
Resting LDF (U)	SHAM LPS	202 ± 56	198 ± 64	186 ± 64	189 ± 72	204 ± 96	215 ± 104	250 ± 105
Resting LDF (U)	VGX LPS	160 ± 70	141 ± 58	151 ± 62	157 ± 57	192 ± 60	198 ± 67	196 ± 59
Resting LDF (U)	VGX LPS + STIM	173 ± 68	165 ± 69	177 ± 69	185 ± 65	227 ± 35	231 ± 56	227 ± 52

Data are given as the mean ± standard deviation (SD) together with statistical differences. Significance is given as: *, *P* < 0.05; **, *P* < 0.01; ***, *P* < 0.001; ^s^, *P* < 0.0001 compared to baseline. BP, blood pressure; EFVR, evoked flow velocity responses; LDF, laser Doppler flowmetry; LPS, lipopolysaccharide; SEP, somatosensory evoked potentials; SHAM, sham surgery; VGX, bilateral vagotomy; VGX + STIM, bilateral vagotomy and distal vagus nerve stimulation.

**Figure 2:**
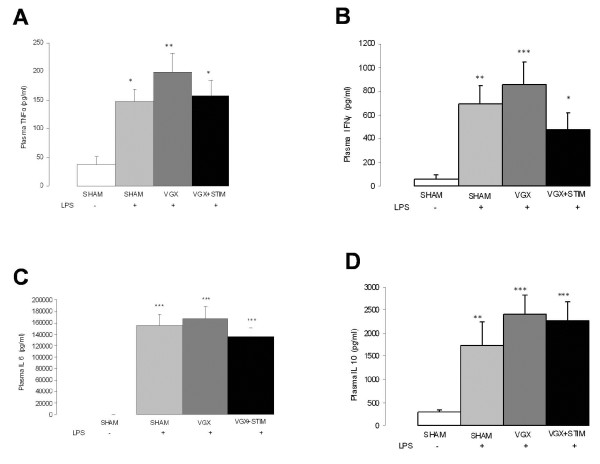
**Cytokine concentrations in plasma samples 4.5 hours after lipopolysaccharide (LPS) administration for all LPS groups and the control (SHAM, white bar) group.** (**A**) TNF-α, (**B**) IFN-γ, (**C**) IL-6, and (**D**) IL-10 levels measured by ELISA. In all groups, a significant increase occurs under septic conditions. Although vagus nerve stimulation (VGX LPS + STIM; black bar) appears to have an anti-inflammatory action, while vagotomy (VGX, gray bar) has a proinflammatory action, statistical examinations between the groups were insignificant. Statistical differences versus SHAM are given as *, *P* < 0.05; **, *P* < 0.01; ***, *P* < 0.001; n = 6 rats each. Data are given as the mean ± SEM.

LPS-treated animals developed signs of a moderate sepsis-like syndrome with typical changes in blood pressure (SHAM LPS: baseline 123 ± 11 mmHg versus 90 ± 18 mmHg, *P* < 0.0001), pH-level (SHAM LPS: baseline 7.47 ± 0.05 versus 7.45 ± 0.03), lactate concentrations (SHAM LPS: baseline 0.8 ± 0.1 mmol/l versus 1.9 ± 0.5 mmol/l, *P* < 0.0001). The data are given in the tables as follows: Table 1 illustrates the group averaged data for glucose, lactate, and pH, partial pressure of oxygen and carbon dioxide as well as hemoglobin content, which was obtained at baseline conditions (baseline) and at the end of the experiment (end). Table 2 shows the group data for mean blood pressure together with N2-P1 potential amplitude, P1 latency, the evoked flow velocity response and resting LDF signal. The cytokines TNF-α, IFN-γ, IL-10, and IL-6 are given in Figure 2.

### Vagal effects in nonseptic rats

In non-endotoxinemic groups, VGX and VNS (VGX + STIM) did not significantly change blood gas, hemodynamic or neurofunctional parameters as compared to the baseline (Tables 1 and 2). Stimulation of the left nervus vagus did not induce a significant decrease in blood pressure. Due to the administration volume of saline, hemoglobin levels decreased moderately until the end of experiments in each group (140 ± 10 g/l versus130 ± 10 g/l and 130 ± 10 g/l versus100 ± 20 g/l, respectively, both *P* < 0.05; Tables 1 and 2).

### Vagal effects in endotoxinemic rats

**Figure 3:**
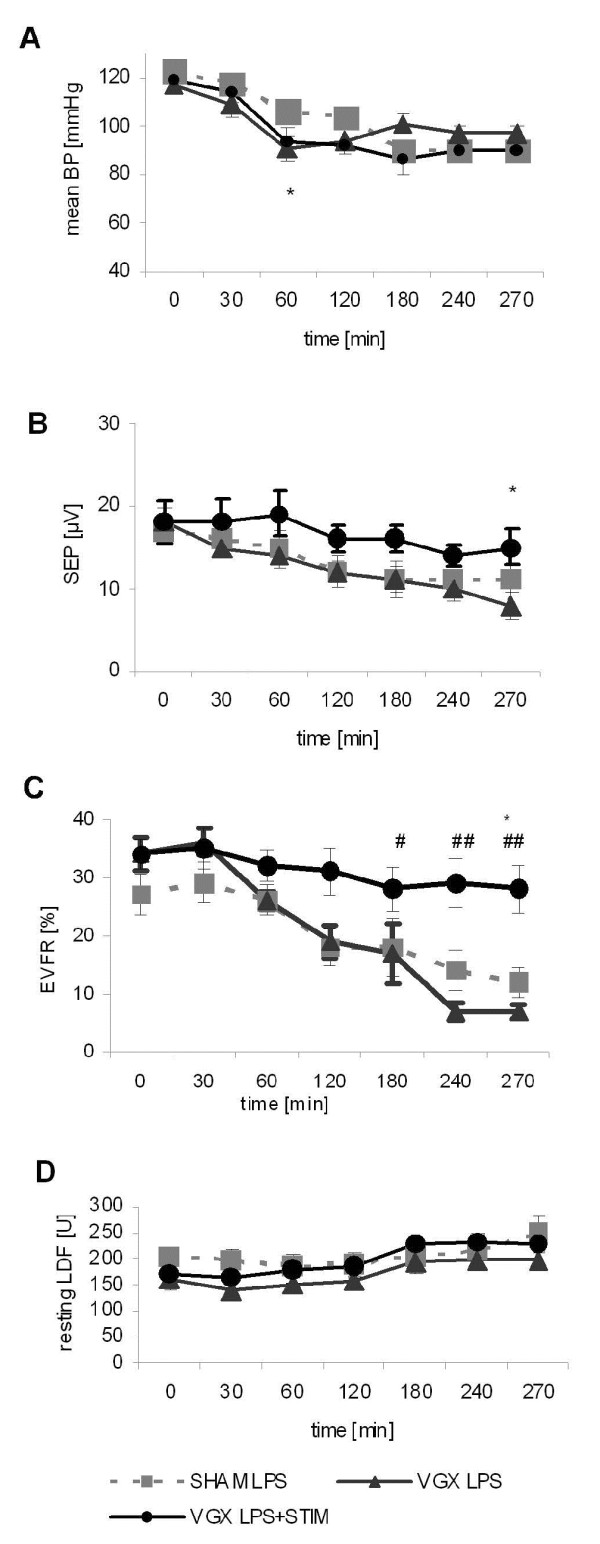
**Data for mean arterial blood pressure (BP, (A)), somatosensory evoked potential amplitudes (SEP, (B)), evoked hemodynamic responses (EVFR, (C)), and resting laser Doppler flow values (resting LDF, (D)) are given for the lipopolysaccharide (LPS)-treated groups.** There were no significant differences for the BP and resting LDF between the groups. The resting LDF increased approximately 30% until the end of experiments. Vagus nerve stimulation stabilized potential amplitudes (VGX LPS + STIM; * *P* < 0.05 versus SHAM LPS, graph B) whereas vagotomy had an opposite effect (VGX LPS) as compared to the sham surgeries (SHAM LPS). The evoked responses remained stable throughout the experiments in the VGX LPS + STIM group showing a significant difference to the SHAM LPS group (*, *P* < 0.05, VGX LPS + STIM versus SHAM LPS, graph C). Again, vagotomy had a negative effect on the uncoupling (^#^, *P* < 0.05, VGX versus VGX LPS + STIM, ^##^, *P* < 0.01, VGX versus VGX LPS + STIM, graph C). n = 10 rats each. Data are given as the mean ± SEM.

Although the changes in hemodynamic and cytokine levels were statistically insignificant between groups, the VNS group tended to mitigate the inflammatory response whereas VGX rats showed a trend toward an increase in inflammatory response. Data for the blood gas analysis, hemodynamic and neurofunctional parameters are given in Tables 1 and 2. VNS or VGX did not have a significant effect on changes in the mean arterial blood pressure (mean BP, Figure 3A) or resting laser Doppler signal (resting LDF, Figure 3D). Both somatosensory evoked potential amplitudes (SEP, Figure 3B) and evoked flow velocity responses (EFVR, Figure 3C), were stabilized under VNS and decreased in VGX (Table 2). In the SHAM LPS group EFVR significantly declined 240 minutes after LPS application with the smallest responses at the end of the experiments (SHAM LPS: 4.5 hours: 12 ± 8% versus baseline: 27 ± 11%; *P* = 0.05). On the one hand, a stronger and earlier (120 minutes) lowering of EFVR was seen in the VGX LPS group (VGX LPS: 4.5 hours: 7 ± 4% versus baseline: 34 ± 9%; *P* < 0.0001). On the other hand, VNS stabilized hemodynamic responses throughout the study (VGX LPS + STIM: 4.5 hours: 28 ± 13% versus baseline: 34 ± 8%; *P* = n.s.). Similarly, VNS stabilized evoked potential amplitudes under inflammatory conditions (VGX LPS + STIM: 4.5 hours: 15 ± 7 μV versus baseline: 18 ± 8 μV; *P* = n.s.) whereas the SHAM LPS group showed a significant decline (SHAM LPS: 4.5 hours: 10 ± 6 μV versus baseline: 17 ± 5 μV; *P* < 0.01). The first significant drop compared to the baseline occurred 240 minutes after LPS application in the SHAM LPS group (Table 2). Again, the VGX LPS group showed a stronger and earlier (120 minutes) significant decline in potential amplitudes (VGX LPS: 4.5 hours: 8 ± 5 μV versus baseline: 18 ± 6 μV; *P* < 0.001). The P1 latencies did not differ with time or between groups (Table 2). The occurrence of cerebral hyperaemia as concluded from the resting flow velocity recordings was approximately 30% among all (Table 2). Comparing the VGX LPS and VGX LPS + STIM group at each time point, we found a significant difference in the EFVR after 180 minutes, whereas the SEP amplitudes first differentiated at 4.5 hours. These data indicate that uncoupling precedes changes in evoked potentials. Similarly, comparing the VGX LPS + STIM and the SHAM LPS groups a significant decline in EFVR in the SHAM LPS group occurred at 4.5 hours, while the potential amplitudes did not differ between the groups at this time point.

### Vagal effects on NO-hypoxia and apoptosis signaling in septic rats

**Figure 4:**
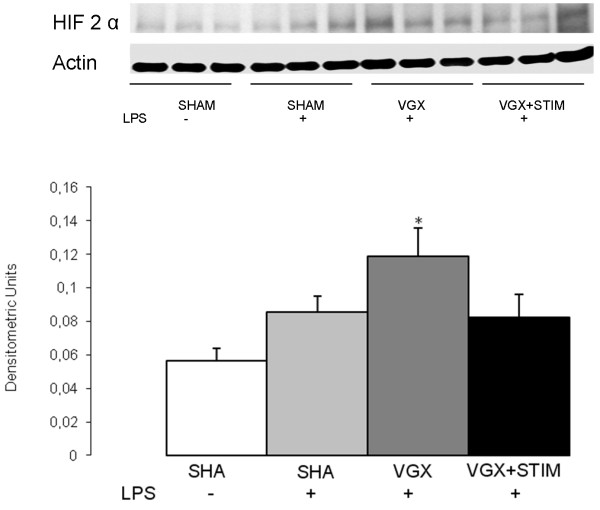
**Western blot analysis and quantification of HIF-2α expression in the cortex 4.5 hours after lipopolysaccharide (LPS) administration for all LPS groups and the control group (SHAM, white bar).** With the exception of the vagotomy group (VGX LPS, gray bar), no significant differences to the SHAM group were found in the LPS-treated and sham-operated (SHAM + LPS, light gray bar) or vagus nerve-stimulated groups (VGX LPS + STIM, black bar). The significant increase in the VGX LPS (gray bar) group is an indicator of a hypoxic condition; * *P* < 0.05 compared to SHAM; n = 6 rats each. Data are given as the mean ± SEM.

**Figure 5:**
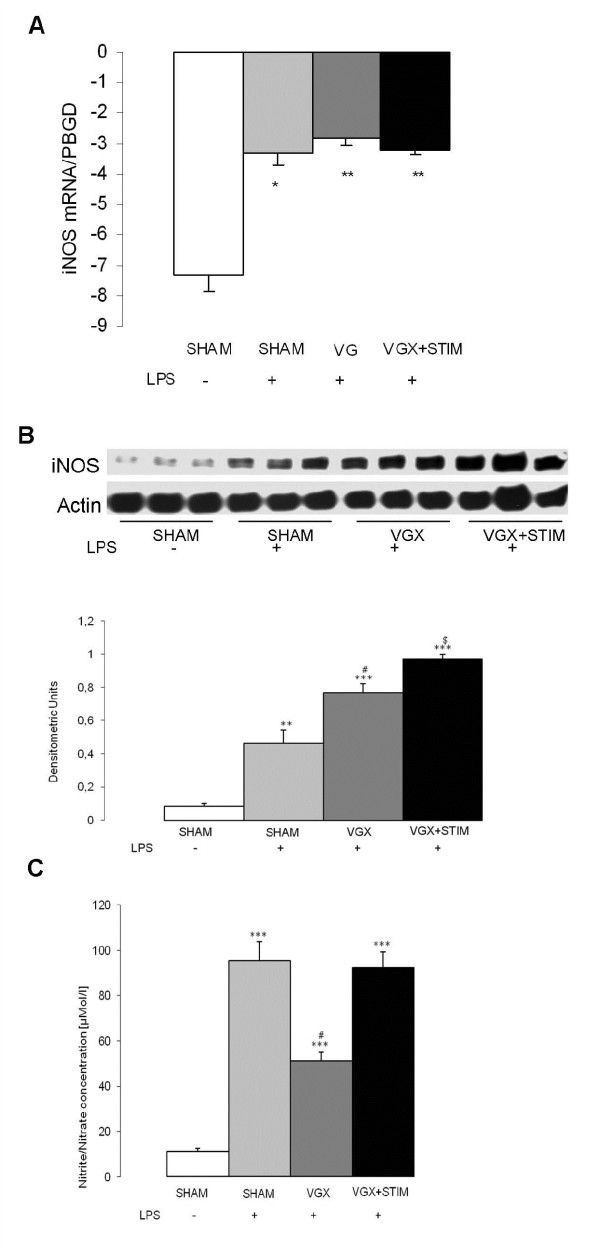
**Real-time PCR iNOS mRNA expression (graph A) and western blot analysis and protein quantification of iNOS expression (graph B) in the cortex 4.5 hours after lipopolysaccharide (LPS) administration for all LPS groups and the control group (SHAM, white bar).** Graph C shows the results of the plasma nitrate/nitrite quantification from samples taken 4.5 hours after LPS administration. Besides a significant increase in the mRNA expression in all LPS groups (*, *P* < 0.05; **, *P* < 0.01 versus SHAM, graph A), there were no differences comparing the vagotomy (VGX LPS, gray bar), LPS-treated and sham-operated (SHAM + LPS, light gray bar) or vagus nerve-stimulated (VGX LPS + STIM, black bar) groups (n = 6 rats). All LPS groups showed a significant increase in iNOS protein compared to SHAM. Within the LPS groups vagotomy (VGX LPS, gray bar) and vagus nerve stimulation (VGX LPS + STIM, black bar) led to a further significant increase compared to SHAM LPS (light gray bar). **, *P* < 0.01, ***, *P* < 0.001 compared to SHAM; ^#^, *P* < 0.01 VGX LPS + STIM versus SHAM LPS; $, *P* < 0.001 VGX LPS + STIM versus SHAM LPS. n = 3 rats each. Nitrite/nitrate significantly increased in all LPS groups compared to SHAM. Compared to SHAM LPS (light gray bar) and vagus nerve stimulation (VGX LPS + STIM) the VGX LPS (gray bar) group showed significant lower values. ***, *P* < 0.001 compared to SHAM; #, *P* < 0.001 compared to SHAM. n = 10 rats each. Data are given as the mean ± SEM.

**Figure 6:**
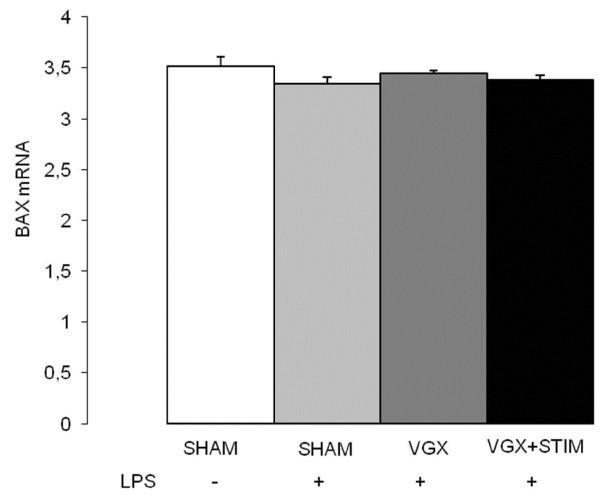
**Real-time PCR BAX mRNA expression in the cortex 4.5 hours after lipopolysaccharide (LPS) administration for all LPS groups and the control group (SHAM, white bar).** Compared to SHAM LPS groups did not show any differences. LPS groups with vagotomy (VGX LPS, gray bar), sham operation (SHAM + LPS, light gray bar) or vagus nerve stimulation (VGX LPS + STIM, black bar) also did not show differences. n = 6 rats each. Data are given as the mean ± SEM.

HIF-2α increased significantly (*P* < 0.05) in the VGX LPS group compared to the SHAM group; no significant changes were found in other LPS groups (Figure 4). Although there were no major differences of iNOS mRNA expression within the LPS groups, there was a significant increase in protein and nitrite/nitrate levels (Figure 5). Interestingly, nitrite/nitrate levels were significantly lower in the VGX LPS group than in other LPS groups (Figure 5). mRNA for the apoptosis signaling molecule BAX did not differ between any groups (Figure 6).

## Discussion

In this study, we demonstrate for the first time that VNS prevented early microvascular dysfunction in the brain of LPS-challenged rats. In contrast, VGX led to an earlier and more pronounced decline in hemodynamic responses. Furthermore, evoked potential amplitudes in VNS animals resulted in a significant protective effect as compared to VGX after 4.5 hours. We speculate that these protective effects might be linked to the anti-inflammatory property of VNS. Conversely, it was shown that increased endotoxin doses resulted in a more severe uncoupling of the NVC relative to the severity of the inflammatory responses [12].

In order to standardize the initial inflammatory response, we chose an intravenous endotoxin model rather than a cecal ligation and puncture model. This issue is crucial in the present study in which the acute changes in evoked potentials and evoked hemodynamic responses were addressed. Furthermore, as animals need no further surgical manipulation, neurophysiological monitoring from stable LDF and electrode positions is guaranteed, enabling a direct, standardized comparison between baseline and inflammatory conditions.

Apoptotic signaling pathways are known to be induced during later phases of sepsis. For example, it has been shown that protein-levels of Bax- and Bcl-related pro- and anti-apoptotic pathways are not induced until eight hours after sepsis induction, with changes in histology only present after twenty-four hours [17]. To confirm our delayed responses we investigated the pro-apoptotic protein Bax at the RNA-level and also did not find a significant change at this early stage.

In contrast to our initial hypothesis, the positive effect on neurovascular coupling cannot be explained by a modification of the NO pathway. The nitrite/nitrate concentrations, as well as the transcription of the iNOS gene, were not affected by VNS. Also, the macrocirculatory parameters did not show typical effects of iNOS inhibition such as an increased blood pressure or prevention of cerebral hyperemia [8]. Moreover, the enzyme levels of iNOS were induced rather than lowered in VNS animals. Although a similar VNS effect was found in the ischemic heart, this issue needs further exploration [18].

Reduced nitrite/nitrate concentrations in the VGX group were unexpected given the pro-inflammatory action of VGX and the increased protein levels of iNOS. Le Cras and McMurty suggested that oxygen substrate limitation may regulate NO production under hypoxic conditions [19]. High altitude-induced pulmonary hypertension is caused by reduced NO production due to a drop in the pO_2_ tension [20]. In stroke models, reduced NOS activity was found in the hypoxic tissue of the penumbra [21]. The dissociation of the microcirculation in areas of hyper- and hypoperfusion and the fluctuation between states hampers direct measurement of oxygen tension in the microcirculation [22]. Taken together, the strong depression of NVC, increased HIF-2α levels, and reduced nitrite/nitrate concentrations are likely due to the brains hypoxic state under VGX. HIF-2α serves as a hypoxia marker under inflammatory conditions since it is not affected by the inflammation itself, such as HIF-1α [23]. Further support of ischemic conditions in the brain during sepsis comes from MRT investigations demonstrating signs of microinfarcts in early phases of sepsis [24]. Sepsis survivors display typical changes in the brain mimicking a hypoxic condition, and are clinically similar to patients who underwent cardiac resuscitation or suffered carbon monoxide intoxication [25-27]. The notion of a local, rather than a general, hypoxic condition in the septic VGX group is supported by blood gas and blood pressure data, which indicate sufficient macrocirculatory and pulmonary functioning.

## Conclusions

Microvascular dysfunction plays an important role in the occurrence of early organ dysfunction in sepsis syndromes. By its anti-inflammatory action, VNS stabilizes the neurovascular coupling as well as evoked potential amplitudes. Further research is needed to explore a similar protective role in other organ systems.

## Abbreviations

BAX, Bcl-2-associated X protein; ECL, enhanced chemiluminescence; EFVR, evoked flow velocity response; ELISA, enzyme-linked immunosorbent assay; iNOS, inducible nitric oxide synthase; IL, interleukin; INF, interferon; LDF, laser Doppler flowmetry; LPS, lipopolysaccharide; NO, nitric oxide; NVC, neurovascular coupling; PBGD, porphobilinogen deaminase; qRT-PCR, quantitative real time polymerase chain reaction; RIPA, radioimmunoprecipitation assay; SEP, somatosensory evoked potentials; STIM, stimulation; TFN, tumor necrosis factor; VGX, vagotomy; VNS, vagus nerve stimulation.

## Competing interests

The authors declare that they have no competing interests.

## Authors’ contribution

SM, AK and BR performed the experiments, data evaluation and wrote the manuscript. KM, RS and SSP, together with SM and AK, performed the western blot, PCR and ELISA experiments and contributed to the data evaluation as well as helped with the drafting of the manuscript. All authors read and approved the final manuscript.
